# Lactic Acid Bacteria Isolated from Human Breast Milk Improve Colitis Induced by 2,4,6-Trinitrobenzene Sulfonic Acid by Inhibiting NF-κB Signaling in Mice

**DOI:** 10.4014/jmb.2303.03018

**Published:** 2023-05-12

**Authors:** Kyung-Joo Kim, Suhyun Kyung, Hui Jin, Minju Im, Jae-won Kim, Hyun Su Kim, Se-Eun Jang

**Affiliations:** 1 Department of Food and Nutrition, Eulji University, Seongnam 13135, Republic of Korea; 2 Department of Research, GREEN CROSS Wellbeing Co., Ltd., Yongin 16950, Republic of Korea; 3 BTC Corporation #906, Technology Development Centre, Ansan 15588, Republic of Korea

**Keywords:** Lactic acid bacteria (LAB), probiotics, inflammatory bowel disease (IBD), colitis, 2,4,6-trinitrobenzene sulfonic acid (TNBS), NF-κB

## Abstract

Inflammatory bowel disease (IBD), a chronic inflammatory disease, results from dysregulation of the immune responses. Some lactic acid bacteria (LAB), including *Lactobacillus*, alleviate IBD through immunomodulation. In this study, the anti-colitis effect of LAB isolated from human breast milk was investigated in a mouse model induced acute colitis with 2,4,6-trinitrobenzene sulfonic acid (TNBS). TNBS remarkably increased weight loss, colon shortening, and colonic mucosal proliferation, as well as the expression levels of inflammatory cytokines, including tumor necrosis factor-alpha (TNF-α) and interleukin (IL)-1β. Oral administration of LAB isolated from human breast milk resulted in a reduction in TNBS-induced colon shortening, as well as induced cyclooxygenase (COX)-2, nitric oxide synthase (iNOS), nuclear factor-kappa B (NF-κB). In addition, LAB suppressed inflammatory cytokines such as TNF-α, IL-6, and IL-1β, and thus showed an effect of suppressing the level of inflammation induced by TNBS. Furthermore, LAB alleviated gut microbiota dysbiosis, and inhibited intestinal permeability by increasing the expression of intestinal tight junction protein including ZO-1. Collectively, these results suggest that LAB isolated from human breast milk can be used as a functional food for colitis treatment by regulating NF-κB signaling, gut microbiota and increasing expression of intestinal tight junction protein.

## Introduction

South Korea became an aged society in 2017, at the fastest rate worldwide, and is expected to become a superaged society by 2025. Aging and inflammation are closely related, and recent research results in animal models showed that aging is a consequence of the accumulation of persistent inflammatory responses [1, 2]. In addition, the imbalance in the intestinal microflora caused by the consumption of a high-fat and high-sugar diet increases the inflammatory response. Therefore, inflammatory diseases have been rapidly increasing in Korea over the past 30 years [3].

Inflammatory bowel disease (IBD) is a representative inflammatory disease that mainly occurs at a young age. It causes defecation-related symptoms and great pain in patients. Furthermore, individuals affected by IBD experience continuous or repeated problems as the disease cannot be cured [4, 5]. Thus, IBD places a heavy burden on patients and society [6].

IBD can be divided into ulcerative colitis (UC) and Crohn's disease (CD), and its cause has not yet been clearly identified [7, 8]. UC is a disease in which ulcers are steadily disposed on the mucous membrane of the large intestine, resulting in bloody stools, diarrhea, and abdominal pain. In gross cases, systemic signs, such as anemia, fever, and weight loss appear. UC can take place anywhere in the gastrointestinal tract. CD is an illness in which injuries, such as ulcers, occur discontinuously in any segment of the digestive tract from the mouth to the anus. Although UC and CD differ in lesions and inflammatory symptoms, they have many similarities; therefore, the distinction between the two diseases is often unclear [9]. Since a radical cure has not been established, therapeutic agents that relieve symptoms are mainly used. However, as these agents, including aminosalicylic acid, exhibit various side effects and symptoms recur when the treatment is stopped, the development of effective and safe therapeutic agents is required [10].

Lactic acid bacteria (LAB) manifest various health-advancing activities, such as maintaining the balance of the intestinal flora, promoting the proliferation of beneficial intestinal bacteria, and aiding immune regulation [11]. Recently, several studies have been conducted to prevent or treat colitis using LAB. Numerous researches have reported that LAB, including *Lactobacillus* and *Bifidobacterium* inhibit the inflammatory responses in dextran sulfate sodium- induced colitis mouse model and 2,4,6-trinitrobenzene sulphonic acid (TNBS)- colitis mouse model as well as in thing [12, 13]. In particular, *L. sakei* regulates the inflammatory response by interfering with the signaling pathway nuclear factor-kappa B (NF-κB) [14], and *L. plantarum* relieves colitis by inducing polarization of M1 to M2 macrophage [15].

In this study, the effects of anti-colitis of LAB isolated from breast milk were evaluated. The possibility of LAB isolated from human breast milk as a treatment for IBD was confirmed, as well as the possibility of breast milk as a source of LAB.

## Materials and Methods

### Materials

Roswell Park Memorial Institute (RPMI) medium, TNBS, lipopolysaccharide (LPS) purified from *Escherichia coli* O111:B were supplied from Sigma-Aldrich (USA). Radioimmunoprecipitation assay (RIPA) buffer used as lysis buffer was supplied from Biosesang (Korea). Enzyme-linked immunoassay (ELISA) kits were supplied from BioTechne (USA) for the detection of interleukin (IL)-1β, tumor necrosis factor-alpha (TNF-α), and IL-6. Antibodies for inducible NO synthase (iNOS), cyclooxygenase (COX)-2, p65 (NF-κB), p-p65 (phosphorylated-NF-κB), and β-actin were procured from Cell Signaling Technology (USA). The TMB-Blotting Solution was supplied from Thermo Fisher Scientific (USA).

### Selection and Culture of LAB

LAB strains selected from Korean human breast milk were grown at MRS broth (BD Biosciences). Human breast milk samples were donated by three Korean healthy women. The subjects were selected as nursing mother with no history of high risk of pregnancy and less than 1 month after childbirth. The selected LAB strains were identified by whole genome analysis. An experiment was prepared to observe the effect of anti-inflammatory activity in the macrophages of LAB. After creating an anaerobic environment at 37°C, LAB were cultured in MRS medium. At 5,000 ×*g* for 20 min, the cultured bacteria were centrifuged, the precipitated pellets were recovered, and washed twice with saline. In case of in vitro assays, the collected bacteria were suspended in 1 ml of saline and then deactivated by incubation at 72°C for 30 min. For in vivo assays, cells were incubated in MRS medium until the optical density reached 1 to 2 at 600 nm, prior to washing with saline, proceed with centrifugation (5,000 ×*g* for 20 min). The pelleted bacterial cells adjusted to a concentration of 1 × 10^9^ colony-forming units were suspended in 1% glucose for oral administration to mice.

### In Vitro Assay

To measure the effect of the LAB candidate group on the amount of TNF-α produced by LPS stimulation, In a 24-well plate, concentration of 1 × 10^6^ cells/well of RAW 264.7 macrophages were dispensed and cultured for 24 h. Each candidate group was treated with LAB (1 × 10^5^ cells/well), and 30 min later, LPS (100 ng/ml) was treated. Cytokine concentration in the cell supernatant was measured after the culturing the cells for 24h [14]. Strains that inhibited TNF-α were subsequently selected and applied in vivo.

### Animals

The 6-week-old ICR mice were supplied from Orient Bio (Korea). Mice breeding proceeded under controlled environmental conditions at 20-22°C temperature, 50 ± 10% humidity, and 12 h cycles for light. A standard experimental feed (Samyang, Korea) was used, and ad libitum access to drinking water was provided. The experiment was conducted after approval by Eulji University's Ethics Committee (EUIACUC21-15) and in accordance with Eulji University's Animal Ethics Regulations.

### Induction of Acute Colitis by TNBS and LAB Administration

After acclimatization to the breeding environment for one week, the experiment was conducted by dividing the animals into normal control (NC), colitis induction (TNBS), LAB-administered, and positive control (sulfasalazine, 50 mg/kg) groups, each having the same average weight of six animals. With TNBS, acute colitis was induced in all experimental animals except for the NC group. First, the experimental animal was anesthetized by inhalation of isoflurane. Then, the TNBS solution (2.5 g) was prepared by mixing with 50% ethanol, and 0.1 ml was administered into the colon through the anus using an 1 ml syringe with a round tip, which was lifted vertically, and held for 30 s to induce inflammation. Mice in the NC group were administered 0.1 ml of physiological saline. Subsequently, the LAB sample was suspended in physiological saline for three days from the next day and orally administered at a predetermined dose. In this study, the breeding and treatment of experimental animals was carried out in accordance with the animal ethics regulations of Eulji University. The degree of colitis was scored according to the presented macroscopic scoring guide: 0, free of ulcer or inflammation; 1, free of ulceration or hyperemia observed in localized areas; 2, ulceration without hyperemia; 3, inflammation and ulceration in one area; 4, two or more areas of inflammatory and ulceration were found ³ 2 sites; 5, ulceration spreading > 2 cm [14]. At a temperature of -80°C, colon tissues were stored until the experiment was completed, and some of which were immersed in 4% paraformaldehyde (PFA) and then tissue staining was performed.

### Assay of Myeloperoxidase (MPO) Activity

To obtain the supernatant, lysis buffer (200 μl) was added to 100 mg of colon tissue, the mixture was finely homogenized and centrifuged at 13000 rpm for 15 min at 4°C. MPO activity was tested in the supernatant using a Mouse Myeloperoxidase DuoSet ELISA kit (BioTechne, USA).

### Measurement of Inflammatory Cytokines and Inhibition of Expression of Inflammatory Response Markers

For immunoblot analysis, 250 μl of RIPA buffer holding a protease inhibitor cocktail was added to 100 mg of colon tissue from the model animal and homogenized. Next, the supernatant obtained through the process of setting it to centrifugation at 13000 ×*g* for 15 min at 4°C was stored at -80°C, and β-actin COX-2, iNOS, p65 and p-p65 expression levels were experimented using western blotting. For 1 h and 30 min, 50 μg of the supernatant was electrophoresed using a 10% (w/v) polyacrylamide gel. Then, the proteins were transferred to a nitrocellulose membranes. Following blocking with the blocking buffer for 30 min, the membrane was washed with PBS-tween 20 for 5 min three times each, and cultured overnight with the primary antibody (Cell Signaling Technology) at a ratio of 1:100. The next day, after washing the membrane three times for 10 min each, secondary antibody was incubated for 1h at a ratio of 1:1000. Prior to visualized of the fluorescent bands, the membrane in the wash step was washed 3 times for 15 min each [14].

For cytokine measurements, supernatants from colonic homogenates and lysates of macrophages were used. Using ELISA kits, the levels of TNF-α, IL-1β, and IL-6 were investigated according to the recommended protocol [16].

### Staining of Colonic Tissue

For hematoxylin-eosin (H&E) staining, colonic tissue was post-fixed by immersion in 4% PFA at 4°C for 2–4 h. The tissue was stored in 30% sucrose, which completely penetrated the tissue. Then, tissue were cut into 10-μm thick sections using a cryostat (Leica, Germany), dyed with H&E, and examined by electron microscope.

### Immunohistochemistry (IHC) Staining

To proceed with IHC staining, colonic tissue was immersed in 4% paraformaldehyde at 4°C for 2~4 h and post-fixation was performed. The tissue was stored in 30% sucrose, which completely penetrated the tissue, and then cut with a cryostat (Leica) to a thickness of 10 μm. IHC was subsequently performed. To check the extent to which tight junction proteins appear, rabbit polyclonal anti-Zo-1 IgG, rabbit polyclonal anti-occludin IgG, and rabbit polyclonal anti-claudin IgG were reacted with the primary antibody. Next, tissue sections were incubated with the secondary antibody and avidin and biotinylated peroxidase complex. The color development of the antibody was induced with 3,3'-diaminobenzidine (DAB, Merck, Germany) using an ABC kit (Vector Laboratories, USA). After drying, the samples were sealed in Permount (Merck) using ethanol and xylene, and observed using an optical microscope (BX60, Olympus Co., Japan).

### Next Generation Sequencing (NGS) Analysis

Feces present in the large intestine of three mice randomly selected from each group were used for the intestinal microbiome analysis. NGS analysis was commissioned by Macrogen, and the relative composition of bacteria in each group was compared and analyzed.

### Cell Culture

The human colon epithelial cell HT-29(KCLB No. 30038) was used to create a model of intestinal mucosa. HT-29 cell line was cultivated in Dulbecco’s modified eagle medium (DMEM, GIBCO) supplemented with 1%penicillin/streptomycin (P/S, GIBCO) and 10% fetal bovine serum (FBS, GIBCO). The cell line was preserved at 37°C in a 5% CO_2_ and 95% air humidified atmosphere, and subcultured at 70 to 80% confluence points.

### Probiotics Adhesion Assay

HT-29 cell was seeded in 24 well plate with a density of 1 × 10^5^ cells/ml and grown to 100% confluence in a humidified atmosphere of at 37°C and 5% CO_2_ (usually 5-7days). Probiotics suspension were then washed two times in PBS (pH 7.4, GIBCO) and finally resuspended in DMEM without antibiotics. Final concentration of probiotics was 1 × 10^9^ CFU/ml. After culturing for 2 h, unattached bacteria were removed by the cell layers were washed two times with PBS to remove . Cell layers were dissolved using of Trypsin 0.25%-EDTA(Biowest). The suspensions containing the attached bacteria were diluted and applied to the MRS agar. Colony forming units cultured under anaerobic conditions at 37°C were counted after 48 h of incubation. Percentage of adhesion was calculated as percentage of the bacteria that were bonded with respect to the total number of bacteria added.

### Statistical Analysis

As mean ± standard deviation, all experimental data are represented . Statistical analysis was performed by performing post-hoc analysis using Dunnett's comparison tests after using one-way ANOVA. Differences *p* < 0.05 were considered statistically significant.

## Results and Discussion

### Selection of LAB That Inhibit Inflammatory Cytokines in LPS-Stimulated RAW 264.7 Macrophages

**Fig. 1:**
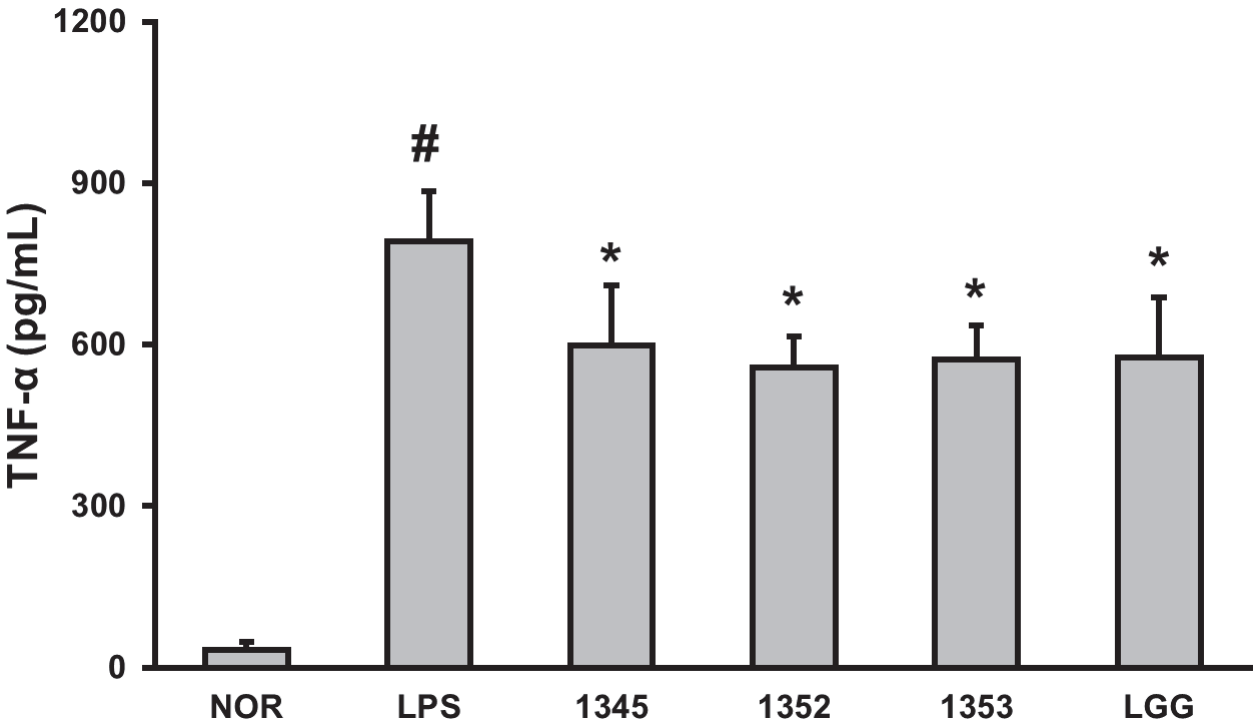
TNF-α inhibitory effect of 3 types of LAB isolated from human breast milk in RAW 264.7 cells treated with LPS. RAW264.7 cells (1 × 10^6^ cells/well) were treated with 100 ng/ml LPS in the presence or absence of heat-inactivated LAB (1 × 10^5^ CFU/well) for 20 h. To determine the level of TNF-α, culture supernatants were used for ELISA. The mean ± SD (*n* = 3) showed enzyme activity values. #Significantly difference are derived compared to normal control group (*p* < 0.05). *Significantly difference are derived compared to LPS-only treated group (*p* < 0.05).

TNF-α is an inflammatory cytokine produced by CD4^+^ CD8^+^ peripheral blood T lymphocytes, monocytes, and macrophages. TNF-α, which closely related to systemic inflammation, plays an important marker for regulating immune cells. To measure the effect of 13 LAB on the amount of TNF-α produced by LPS stimulation, In a 24-well plate, RAW 264.7 macrophages were aliquoted at a concentration of 1 × 10^6^ cells/well and grown for 24 h. As a positive control, *L. rhamnosus* LGG was used. Each of the 13 LAB (1 × 10^5^ cells/well) was added to the cells and 30 min later, LPS (100 ng/ml) was added. Subsequently, culturing the cells for 24 h, the cytokine concentration was measured using the cell supernatant. Three strains that significantly reduced the TNF-α levels following LPS treatment were selected (Fig. S1), and reconfirm (Fig. 1). As a result of whole genome analysis, GCWB1345 was identified as *Bifidobacterial breve*, GCWB1352 as *L. rhamnosus*, and GCWB1353 as *Lactobacillus* paragasseri.

### LAB Isolated from Human Breast Milk Ameliorates TNBS-Induced Colitis in Mice

A TNBS-induced colitis mouse model was used to confirm the anti-colitis effect of LAB isolated from breast milk. In general, experimental models of TNBS-induced colitis are used to study the incidence of IBD in animals [17]. TNBS-induced colitis, which is being used as a useful model to investigate the effectiveness of various new anti-inflammatory treatments, as it positively responds to anti-TNF-α antibody therapy and other currently available IBD treatments such as sulfasalazine [18, 19].

**Fig. 2:**
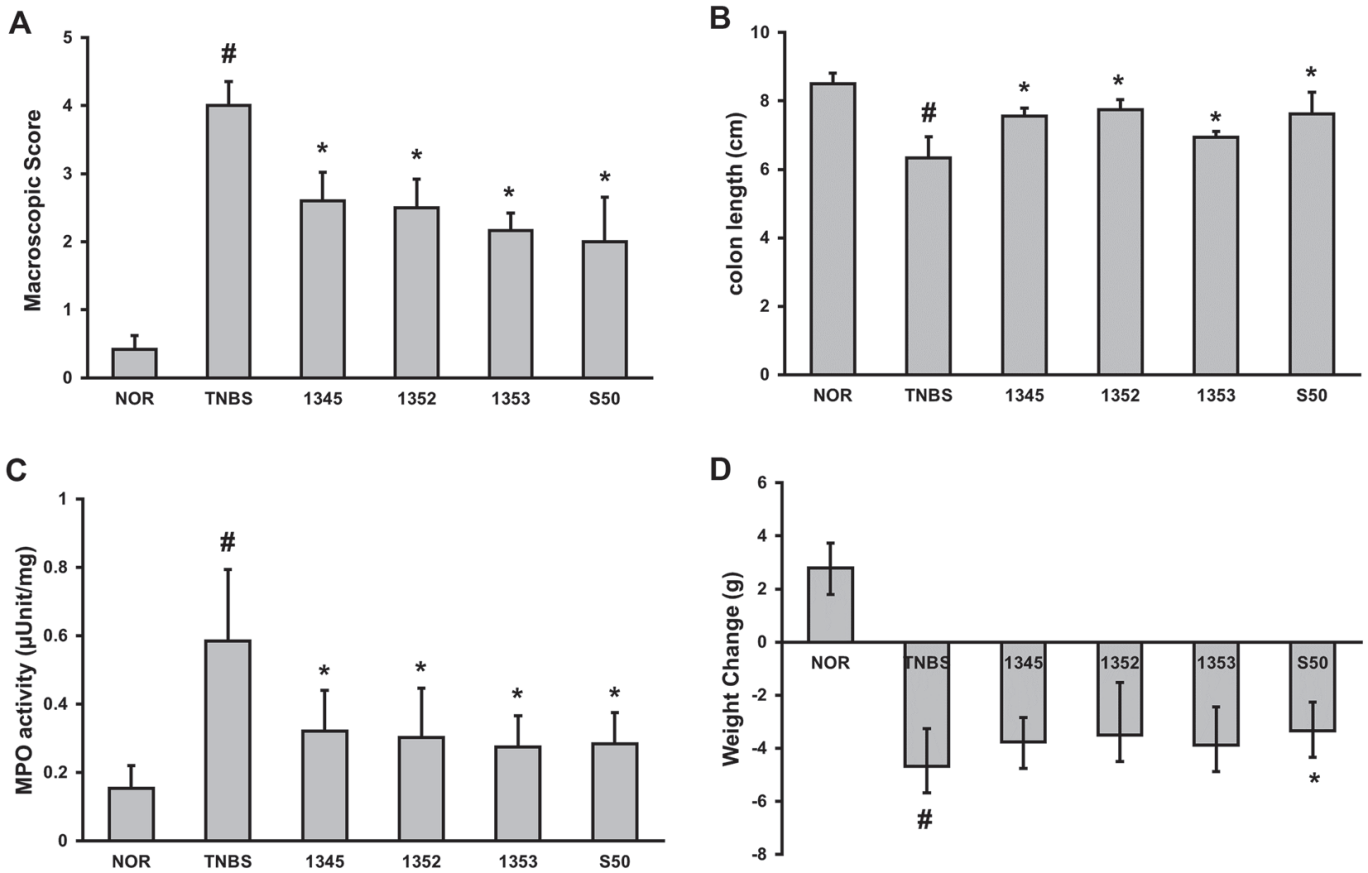
Effects of GCWB1345, GCWB1352 and GCWB1353 in TNBS-induced colitis mice. (**A**) Macroscopic score. (**B**) Effect on colon length. (**C**) Effect on MPO activity (**D**) Effect on weight change. TNBS was administered intrarectally to each of the TNBS, GCWB1345, GCWB1352, GCWB1353, and S50 groups. After TNBS injection, the LAB (TNBS, saline only; 1345, 1 × 10^9^ CFU/mouse of GCWB1345; 1352, 1 × 10^9^ CFU/mouse of GCWB1345; 1353, 1 × 10^9^ CFU/mouse of GCWB1345; S50, 50 mg/kg sulfasalazine) were 3 days of oral administration. Normal control (Nor) was treated with vehicle instead of LAB. All experimental values are expressed as mean ± SD (*n* = 6). ^#^Significantly difference are derived compared to normal control group (*p* < 0.05). *Significantly difference are derived compared to TNBS-only treated group (*p* < 0.05).

In the TNBS-treated negative control group, the length of the intestine was shortened and inflammation was observed comparison with the normal control group, whereas in the group administered the selected three LAB, the length of the intestine was longer and the symptoms of colitis were alleviated compared to the negative control group (Figs. 2A and 2B). In addition, MPO, an enzyme that catalyzes the production of reactive oxygen species, known as a biomarker of oxidative damage, was confirmed to limit by three LABs selected from breast milk (Fig. 2C).

**Fig. 3:**
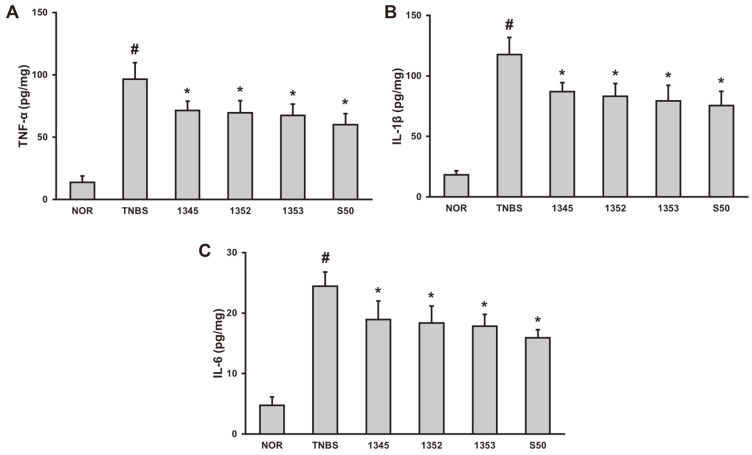
Effects of GCWB1345, GCWB1352, and GCWB1353 on pro-inflammatory cytokines TNF-α (A), IL-1β (B), and IL-6 (C) in TNBS-induced colitis mice. TNBS was administered intrarectally to each of the TNBS, GCWB1345, GCWB1352, GCWB1353, and S50 groups. After TNBS injection, the LAB (TNBS, saline only; 1345, 1 × 10^9^ CFU/ mouse of GCWB1345; 1352, 1 × 10^9^ CFU/mouse of GCWB1345; 1353, 1 × 10^9^ CFU/mouse of GCWB1345; S50, 50 mg/kg sulfasalazine) were 3 days of oral administration. Normal control (Nor) was treated with vehicle instead of LAB. All experimental values are expressed as mean ± SD (*n* = 6). ^#^Significantly difference are derived compared to normal control group (*p* < 0.05). *Significantly difference are derived compared to TNBS-only treated group (*p* < 0.05).

**Fig. 4:**
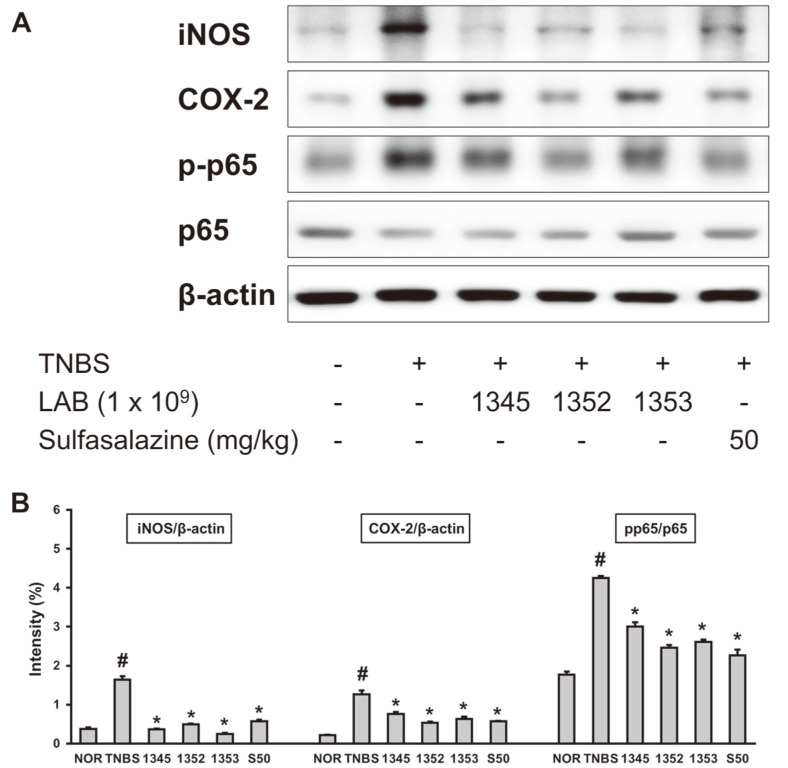
Effect of GCWB1345, GCWB1352, and GCWB1353 on NF-κB in TNBS-induced colitis mice. TNBS was administered intrarectally to each of the TNBS, GCWB1345, GCWB1352, GCWB1353, and S50 groups. After TNBS injection, the LAB (TNBS, saline only; 1345, 1 × 10^9^ CFU/mouse of GCWB1345; 1352, 1 × 10^9^ CFU/mouse of GCWB1345; 1353, 1 × 10^9^ CFU/mouse of GCWB1345; S50, 50 mg/kg sulfasalazine) were 3 days of oral administration. Normal control (Nor) was treated with vehicle instead of LAB. The protein levels were measured by immunoblotting. ^#^Significantly difference are derived compared to normal control group (*p* < 0.05). *Significantly difference are derived compared to TNBS-only treated group (*p* < 0.05).

Inflammatory cytokines are involved in the early inflammatory response in colitis. In particular, the increased production of TNF-α induces the death of epithelial cells, destroying the mucosal layer and continuously triggering an inflammatory response [20]. The amount of TNF-α, IL-1β, and IL-6 in lysed intestinal tissue solution were determined. Compared to the normal control group, the expression of inflammatory cytokines including TNF-α were significantly increased in the negative control group, and the expression level of inflammatory cytokines were significantly decreased when the results of oral administration of the selected LAB were compared to the negative control group (Fig. 3). In addition, COX-2 and iNOS, proteins upregulated during the inflammatory response, were evaluated in intestinal lysates by immunoblot. The three selected LABs significantly lowered the expression levels of these proteins compared to the negative control group (Fig. 4).

The ubiquitous transcription factor NF-κB determines various cellular roles such as inflammatory response, proliferation and adhesion [21]. NF-κB, normally present in the cytoplasm, is an inactive heterodimer consisting of the p50 and p65 (RelA) subunits, is stimulated by endotoxins such as LPS and then moved to the nucleus. NF-κB (pp65) begins to be activate in the nucleus ties to appropriate sites in the DNA to induces the activity of TNF-α and IL-1β [22]. The Activation mechanism of NF-κB is achieved through a positive feedback mechanism of pro-inflammatory cytokines [23]. Therefore, in IBD, NF-κB signaling should be inhibited as much as possible. LAB isolated from breast milk strongly inhibited the expression of NF-κB induced by TNBS (Fig. 4). This result shows that the various inflammatory cytokine inhibitory effects of the selected LAB are due to inhibition of NF-κB.

**Fig. 5:**
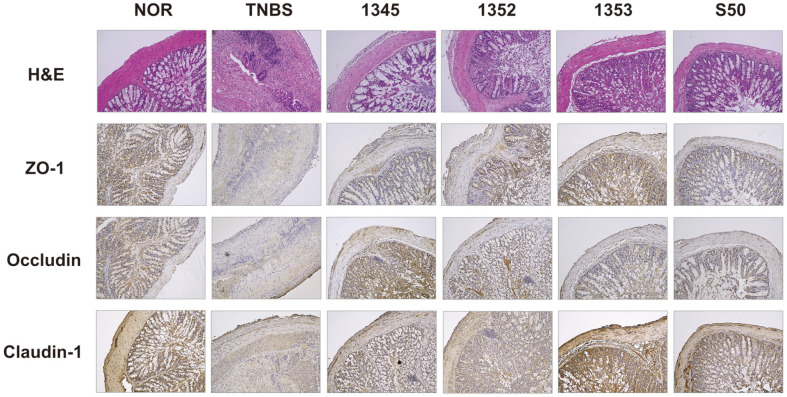
Effects of GCWB1345, GCWB1352 and GCWB1353 on the expression of intestinal tight junction protein in TNBS-induced colitis mice. Histological examination of colon tissues, stained with hematoxylin-eosin and immunostained with anti-Zo-1, anti-occludin, and anti-claudin antibody. TNBS was administered intrarectally to each of the TNBS, GCWB1345, GCWB1352, GCWB1353, and S50 groups. After TNBS injection, the LAB (TNBS, saline only; 1345, 1 × 10^9^ CFU/mouse of GCWB1345; 1352, 1 × 10^9^ CFU/mouse of GCWB1345; 1353, 1 × 10^9^ CFU/mouse of GCWB1345; S50, 50 mg/kg sulfasalazine) were 3 days of oral administration. Normal control (Nor) was treated with vehicle instead of LAB. All experimental values are expressed as mean ± SD (*n* = 6). ^#^Significantly difference are derived compared to normal control group (*p* < 0.05). *Significantly difference are derived compared to TNBS-only treated group (*p* < 0.05).

Analysis of the degree of tissue inflammation using H&E staining presented that the histological damage was remarkably reduced in the group administered with the selected LAB compared to the TNBS group. Typical inflammatory changes in colon tissue, such as colonic mucosa thickening and inflammatory cell infiltration, were clearly observed in the TNBS group than in the normal control group. In contrast, normal colonic tissue morphology was observed following administration of LAB, which counteracted the histological characteristics of TNBS-induced colitis (Fig. 5A). These results suggest that LAB administration reduces the proliferation of TNBS-induced epithelial cells of the colonic mucosa.

When the tight junction of the intestine is weakened, permeability increases [24]. Since colitis is a representative disease in which intestinal permeability is increased, proteins related to intestinal compaction were analyzed using IHC. The analysis showed that the group administered LAB isolated from breast milk had a tendency to increase the expression of proteins related to dense bonding compared to the TNBS group. In the TNBS group, compared to the normal control group, the expression of ZO-1, occludin, and claudin 1, proteins related to dense junctions, was significantly decreased, but administration of LAB restored the TNBS-induced decrease in dense junction proteins (Fig. 5B). These results suggest that the administration of LAB increased the tight-binding protein levels that were reduced by TNBS.

### LAB Isolated from Human Breast Milk Changes Composition of Intestinal Microflora

**Fig. 6:**
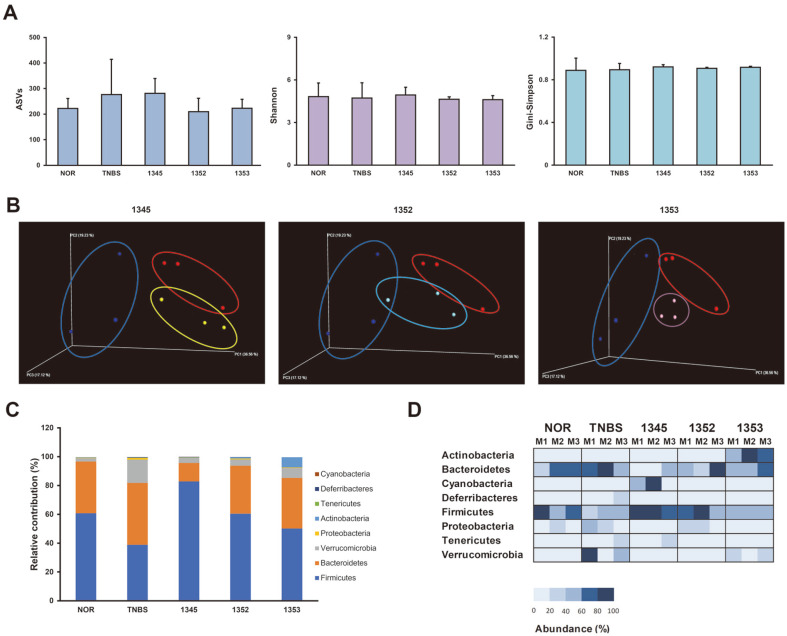
Effect of GCWB1345, GCWB1352, and GCWB1353 on intestinal microbiome composition in mice with TNBS-induced colitis. Effects on the α-diversity (**A**), β-diversity (Normal, blue; TNBS, red; GCWB1345, yellow; GCWB1352, cyan; GCWB1353, purple) (**B**). Effects on the composition at the phylum level (**C**) and (**D**).

While performing NGS analysis of 416.2 M size, the effect of LAB on the microbiome was examined, and the results are shown in each figure. As a result of diversity analysis based on the amplicon sequence variant obtained through taxonomic analysis, changes in the gut microbiome were observed according to the administration of each strain through β-diversity, but not α-diversity (Figs. 6A and 6B). When the composition of the intestinal microbiota was analyzed by group, changes were captured at the phylum level following the administration of LAB. In particular, the decrease in *Firmicutes* with the administration of TNBS showed a tendency to increase with the administration of each strain (Fig. 6C). By analyzing the abundance according to the administration of LAB at the phylum level in mice, the changes in abundance were confirmed. In particular, the abundance of *Firmicutes* decreased and that of *Proteobacteria* increased following the administration of TNBS. However, the administration of LAB isolated from breast milk reversed these changes (Fig. 6D). These results indicate that LAB isolated from human breast milk improves colitis by inducing changes in the gut microbiota.

### Possibility of Developing Functional Foods to LAB Isolated from Human Breast Milk for the Improvement and Prevention of IBD

**Fig. 7:**
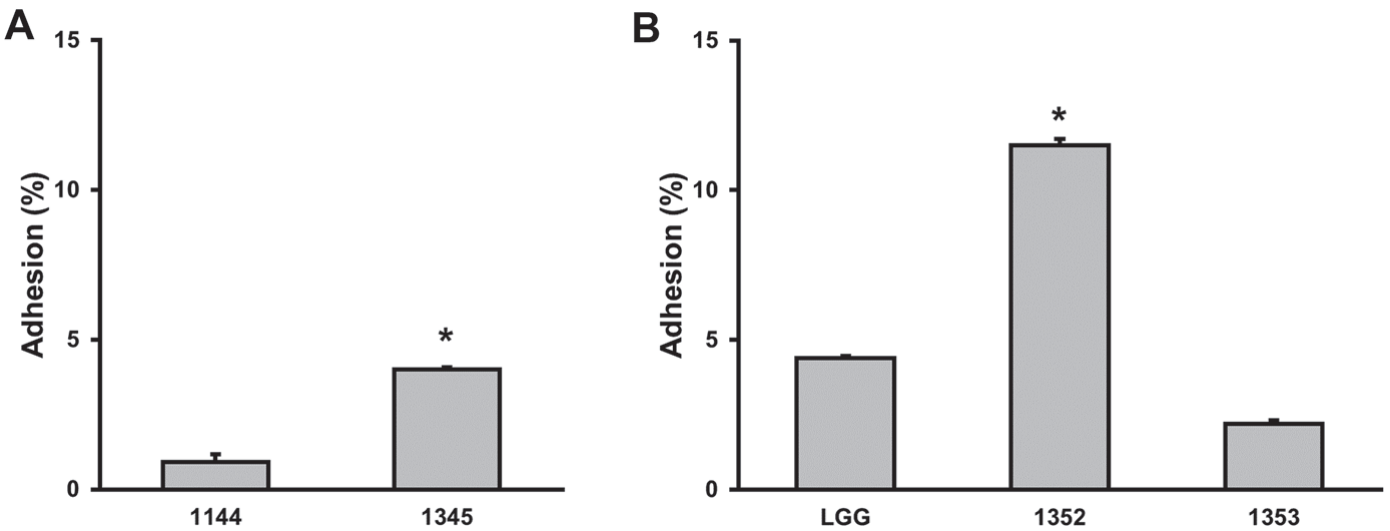
Intestinal adhesion of GCWB1345, GCWB1352, and GCWB1353 in Caco2 cells. Effect of GCWB1345 (**A**), GCWB1342 and GCWB1353 (**B**). *Significantly difference are derived compared to *B. breve* GCWB1144 or *L. rhamnosus* GG treated group (*p* < 0.05).

To evaluate the possibility of developing functional food of the isolated LAB, adhesion assay to human intestinal epithelial cell was conducted. In general, because the adhesion of *Lactobacillus* and *Bifidobacterium* differs depending on the strain [25, 26], LAB of the same species was used as a control. GCWB1345 exhibited high adhesion compared to the human fecal reference strain *B. breve* GCWB1144 (Fig. 7A). This result is consistent with previous studies showing high adhesion capacity of human milk-derived LAB [27]. In addition, GCWB1352 showed significantly higher adhesion than *L. rhamnosus* GG, which is known to have good adhesion to intestinal epithelial cell [28], but the other strain GCWB1353 showed lower adhesion than *L. rhamnosus* GG (Fig. 7B).

In assays to estimate the potential risk of LAB isolated from human breast milk, negative results were obtained (Table S1) except for the MIC assay. In the MIC results, streptomycin for GCWB1345, kanamycin for GCWB1352, and gentamycin for GCWB1353 were higher than the EFSA cut-off values (Table S2). In whole genome sequencing to confirm antibiotic resistance, there were no antibiotic resistance genes except for GCWB1345. GCWB1345 had a tetracycline resistance gene, but it was not acquired resistance (data not shown). These results mean that LAB isolated from human breast milk can be safe in the body.

These results show the possibility LAB isolated from human milk develop to functional foods for the improvement and prevention of IBD by their superior adhesion capacity to human intestinal epithelial cell and safety, and that human milk can be used as a source of functional LAB.

Complex intestinal mucosal immune responses to resident intestinal microbial communities in vivo appear to lead to the onset of IBD [29]. This study demonstrated that the inhibition of NF-κB pathway stimulation by endotoxins produced by gut microbes such as LPS is closely related to alleviation of IBD. LAB isolated from breast milk have the potential to improve colitis through a mechanism that blocking the NF-κB pathway. In addition, administration of LAB significantly increased tight junction proteins and altered the flora of the TNBS group. In addition, LAB isolated from breast milk have excellent adhesion to human intestinal epithelial cell and excellent safety.

These results suggest that LAB isolated from breast milk have anti-colitis effects and potential for human trials. Thus, the development of LAB as a functional food for preventing or improving colitis can be expected.

## Supplemental Materials

Supplementary data for this paper are available on-line only at http://jmb.or.kr.
