# Supplementary material for: Lactic Acid Bacteria Isolated from Human Breast Milk Improve Colitis Induced by 2,4,6-Trinitrobenzene Sulfonic Acid by Inhibiting NF-κB Signaling in Mice
Source: J Microbiol Biotechnol. 2023 May 12;33(8):1057–65. doi: 10.4014/jmb.2303.03018 (PMC10468674; doi:10.4014/jmb.2303.03018)
Supplement: Supplementary file 1 [file jmb-33-8-1057-supple.pdf]

J. Microbiol. Biotechnol.
https://doi.org/10.4014/jmb.2303.03018

Supplementary Figure and Tables

Lactic Acid Bacteria Isolated from Human Breast Milk Improve Colitis Induced by 2,4,6-Trinitrobenzene Sulfonic Acid by Inhibiting NF-κB Signaling in Mice

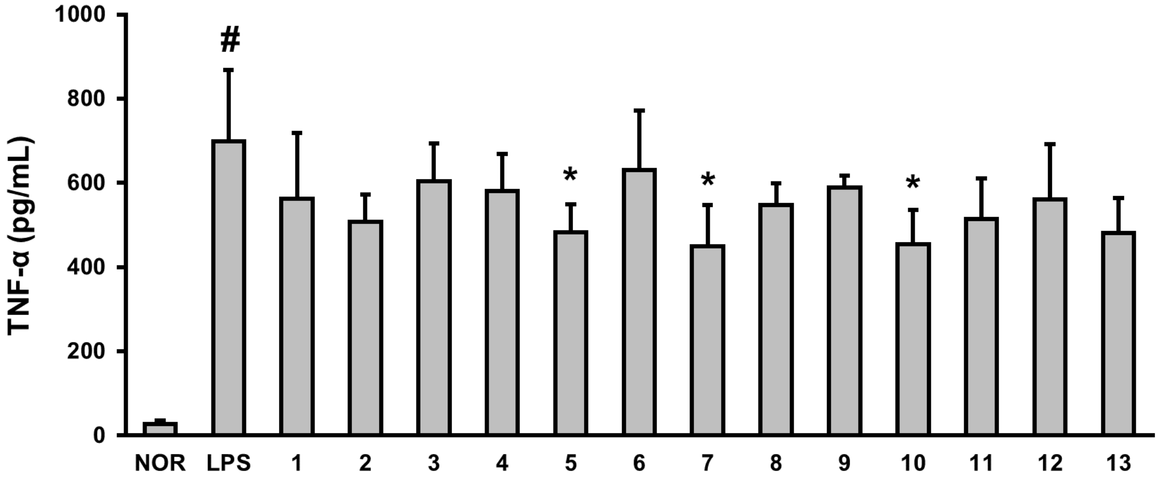

Supplementary Figure S1.

TNF-α inhibitory effect of 13 types of LAB isolated from human breast milk in RAW 264.7 cells treated with LPS.

Supplementary Table S1. Safety assessment.

LAB	Hemolysis[1)]	Cytotoxicity to HT-29[2)]	Bile Salt Hydrolyzing (BSH) activity	D-lactate formation
GCWB1345	γ	ND[3)]	N[4)]	ND
GCWB1352	γ	ND	N	ND
GCWB1353	γ	ND	N	ND

[1)]Type of Hemolysis. α; α-hemolysis, β; β-hemolysis, γ; γ-hemolysis
[2)]The human colon cancer cell line
[3)] ND; Not detected
[4)]Results of incubation at 0.5% Taurodeoxycholic acid in MRS agar. P; positive, N; negative, -; no growth

Supplementary Table S2. Results of MIC test.

GCWB1345 (*Bifidobacterium breve*)									
	Amp	Van	Gen	Kana	Strep	Eryth	Clinda	Tetra	Chlor
1345	0.5	0.75	64	n.r.[2)]	>256*	0.047	0.016	6	0.75
EFSA[1)] breakpoint	2	2	64	n.r.	128	1	1	8	4
GCWB1352 (*Lacticaseibacillus rhamnosus*)									
	Amp	Van	Gen	Kana	Strep	Eryth	Clinda	Tetra	Chlor
1352	0.75	n.r.	16	128*	32	0.25	0.19	0.5	4
EFSA breakpoint	4	n.r.	16	64	64	1	4	8	4
GCWB1353 (*Lactobacillus paragasseri*)									
	Amp	Van	Gen	Kana	Strep	Eryth	Clinda	Tetra	Chlor
1353	0.5	1.5.	24*	16	8	0.094	4	0.38	4
EFSA breakpoint	1	4	4	16	8	1	4	2	4

AMP; ampicillin, Van; vancomycin, Gen; gentamicin, Kana; kanamycin, Step; streptomycin, Eryth; erythromycin, Clinda; clindamycin, Tetra; tetramycin, Chlor; chloramphenicol

[1)]European Food Safety Authority,2018

[2)]negative result

*Resistance
